# Green ternary composite of graphitic carbon nitride/TiO_2_/ polyorthoanisidine for the enhanced photocatalytic treatment of Direct Red 28 for industrial water treatment solutions

**DOI:** 10.3389/fchem.2024.1411980

**Published:** 2024-09-27

**Authors:** Ali S. Alkorbi, Nouman Gill, Muhammad Naveed Anjum, Muhammad Jawwad Saif, Mirza Nadeem Ahmad, Muhammad Bilal Qadir, Zubair Khaliq, Mohd Faisal, Mohammed Jalalah, Farid A. Harraz

**Affiliations:** ^1^ Department of Chemistry, Faculty of Science and Arts at Sharurah, Najran University, Sharurah, Saudi Arabia; ^2^ Department of Applied Chemistry, Government College University, Faisalabad, Pakistan; ^3^ Department of Textile Engineering, National Textile University, Faisalabad, Pakistan; ^4^ Department of Materials, National Textile University, Faisalabad, Pakistan; ^5^ Department of Chemistry, Faculty of Science and Arts, Najran University, Najran, Saudi Arabia; ^6^ Advanced Materials and Nano-Research Centre (AMNRC), Najran University, Najran, Saudi Arabia; ^7^ Department of Electrical Engineering, College of Engineering, Najran University, Najran, Saudi Arabia

**Keywords:** graphitic carbon nitride (g-C_3_N_4_), titanium dioxide (TiO_2_), polyorthoanisidine (POA), photocatalytic degradation, direct red 28, water pollution

## Abstract

Industrial dye effluent causes significant risks to the environment. The present study was focused on photocatalytic degradation of the dye Direct Red 28 using a ternary composite of graphitic carbon nitride, TiO_2_, and polyorthoanisidine (g-C_3_N_4_/TiO_2_/POA), prepared by *in-situ* oxidative polymerization *o-*anisidine. The synthesized composite g-C_3_N_4_/TiO_2_/POA properties were characterized using different analytical techniques. X-ray diffraction (XRD) results revealed the prominent pattern of TiO_2_ and g-C_3_N_4_ in the composite peak at 2θ° while Fourier transform infrared (FTIR) results provided the confirmation peaks for g-C_3_N_4_/TiO_2_/POA and POA at 1,110 cm^−1^ and 1,084 cm^−1^ for C-O-C ether. Scanning electron microscopy (SEM) demonstrated an increase in the average size of the composite up to 428 nm. The energy-dispersive X-ray spectroscopy (EDX) spectrum provided the weight percentages of the C, O, and Ti in the composite were 8.5%, 45.69%, and 45.81%, respectively. The photocatalytic degradation of Direct Red 28 dye under UV irradiation using a composite showed that 86% Direct Red 28 dye was degraded by a 30 mg/L dose of g-C_3_N_4_/TiO_2_/POA in 240 min at pH 2. After four consecutive cycles, the utilized composite showed 79% degradation of Direct Red 28, demonstrating the stability and effectiveness of the g-C_3_N_4_/TiO_2_/POA photocatalyst. The high reusability and efficiency of the g-C_3_N_4_/TiO_2_/POA composite are due to increased light absorption range and reduced e^−^/h^+^ recombination rate in the presence of g-C_3_N_4_ and POA.

## 1 Introduction

Water pollution is increasing due to domestic and industrial wastewater and has attracted much attention from researchers and scientists in recent years. Researchers are trying to develop efficient ways and materials to clean wastewater and seawater to obtain pollution-free water (Liu et al., 2008; Zheng et al., 2020; Xue et al., 2023; Feng et al., 2024; Yu et al., 2024). Industries release hazardous waste, including dyes, fertilizers, pesticide residues, high-pH waste, heavy metals, organic compounds, etc., which contaminate the environment (Panigrahi and Santhoskumar, 2020). Dyes and pigments are the main cause of water pollution. Industrial effluent discharge in stream water is problematic for aquatic life and environmental pollution (Marungrueng and Pavasant, 2006). Dye molecules in water prevent sunlight from affecting aquatic life and ultimately quench photosynthesis (Saleh et al., 2019). The direct contact of dyes with the skin causes skin infections and breathing problems and may be carcinogenic (Elbadawy et al., 2021). Pollutants like medicines and industrial dyes are toxic to the ecosystem and contaminate groundwater supplies. Removing these toxic organic and inorganic pollutants from wastewater has become a critical concern for researchers (Khan and Malik, 2014; Mahmood et al., 2022; Mahmood et al., 2024).

Advanced oxygen process (AOP), the membrane process, adsorption, coagulation, biodegradation, and photocatalytic degradation are some techniques used to remove dyes from contaminated water. Each of these techniques has certain benefits or drawbacks. Because advanced oxidation ensures the complete degradation of toxic compounds to non-toxic products like H_2_O and CO_2_, AOP seems promising among these techniques because it requires no external oxidant (Athanasekou et al., 2018). It has been reported to be an effective method for the degradation of soluble organic contaminants present in water and soil (Jamil et al., 2020). Photocatalytic AOPs use illuminated heterogeneous catalysts and produce charge carrier oxygen species upon the irradiation of light, such as hydroxyl radicals (·OH) and superoxide ions (O_2_)^−^, which act as powerful oxidizing agents (Wang et al., 2023).

TiO_2_ is an effective photocatalyst that uses reactive oxygen species (ROS) to oxidize almost all organic pollutants, and it is widely used for energy and environmental applications (Qadir et al., 2015; Qadir et al., 2016; Athanasekou et al., 2018; Ahmad et al., 2021). The primary benefit of the photocatalytic reaction is its inherent degrading nature; it eliminates the need for mass transfer, permits operation in ambient conditions (where atmospheric oxygen is sufficient as an oxidant), and ultimately has the potential to convert organic carbon into CO_2_. Moreover, a nanostructured TiO_2_ photocatalyst exhibits relatively high chemical stability and is inexpensive and non-toxic. Also, TiO_2_ has a wide band gap of approximately 3.2 eV, which makes it a very effective photocatalyst for UV radiation (Wahi et al., 2005).

Graphitic carbon nitride (g-C_3_N_4_) is an efficient photocatalyst because it can be activated by visible light to generate ROS that degrade organic pollutants (Shakoor et al., 2024). To enhance its photocatalytic potential, researchers merge it with metal to form a cocatalyst, as demonstrated by the previously reported g-C_3_N_4_/TiO_2_ photocatalyst (Sun et al., 2015). This merging decreases the recombination rate of e−/h+ couples of g-C_3_N_4_ and increases the formation of radical species, accelerating photocatalysis (Zhao et al., 2012). Moreover, a recent study reported that composites could decrease the recombination rate e^−^/h^+^ of different photocatalysts to enhance their photodegradation (Anjum et al., 2023; Jalalah et al., 2023; Ganesan and Xanvier, 2024).

The present study was planned to decrease the recombination rate of e^−^/h^+^ couples of g-C_3_N_4_ by making a g-C_3_N_4_, TiO_2,_ and conductive polymer ternary composite. For this purpose, poly(o-anisidine) conductive polymer was used to produce a g-C_3_N_4_/TiO_2_/POA ternary composite due to its ability to increase the e^−^/h^+^ separation and improve photocatalytic activity. Poly(o-anisidine) is preferred due to its ecological stability and simple manufacturing (Alenizi et al., 2019). The structural morphology of these ternary composites was evaluated by Fourier transform infrared (FTIR), scanning electron microscopy (SEM), X-ray diffraction (XRD), energy-dispersive X-ray spectroscopy (EDX), UV-visible spectroscopy, and ImageJ software for the composite particle size analysis. The photocatalytic degradation potential of these ternary composites was evaluated against Direct Red 28 dye.

## 2 Experimental section

### 2.1 Chemical and reagents

Anatase TiO_2_, urea (CH_4_N_2_O, 99%), ethanol (C_2_H_6_O, 95%), sodium hydroxide (NaOH, 97%) hydrochloric acid (HCl, 99%), o-anisidine monomer (CH_3_OC_6_H_4_NH_2_, 99.5%), ammonium persulfate ((NH_4_)_2_S_2_O_8_), and 4,4′-diaminodiphenylamine sulfate hydrate (C_12_H_15_N_3_O_4_S, 99%) were purchased from Aladdin Reagent Co., Ltd. (Shanghai, China). Direct Red 28 (C_32_H_22_N_6_Na_2_O_6_S_2_, 89%) dye was purchased from Tianjin Tianhe Chemical Reagent Factory (Tianjin, China). All experiments used deionized (DI) water. These substances are all utilized without purification because they are all analytically pure.

### 2.2 Synthesis of g-C_3_N_4_

**FIGURE 1:**
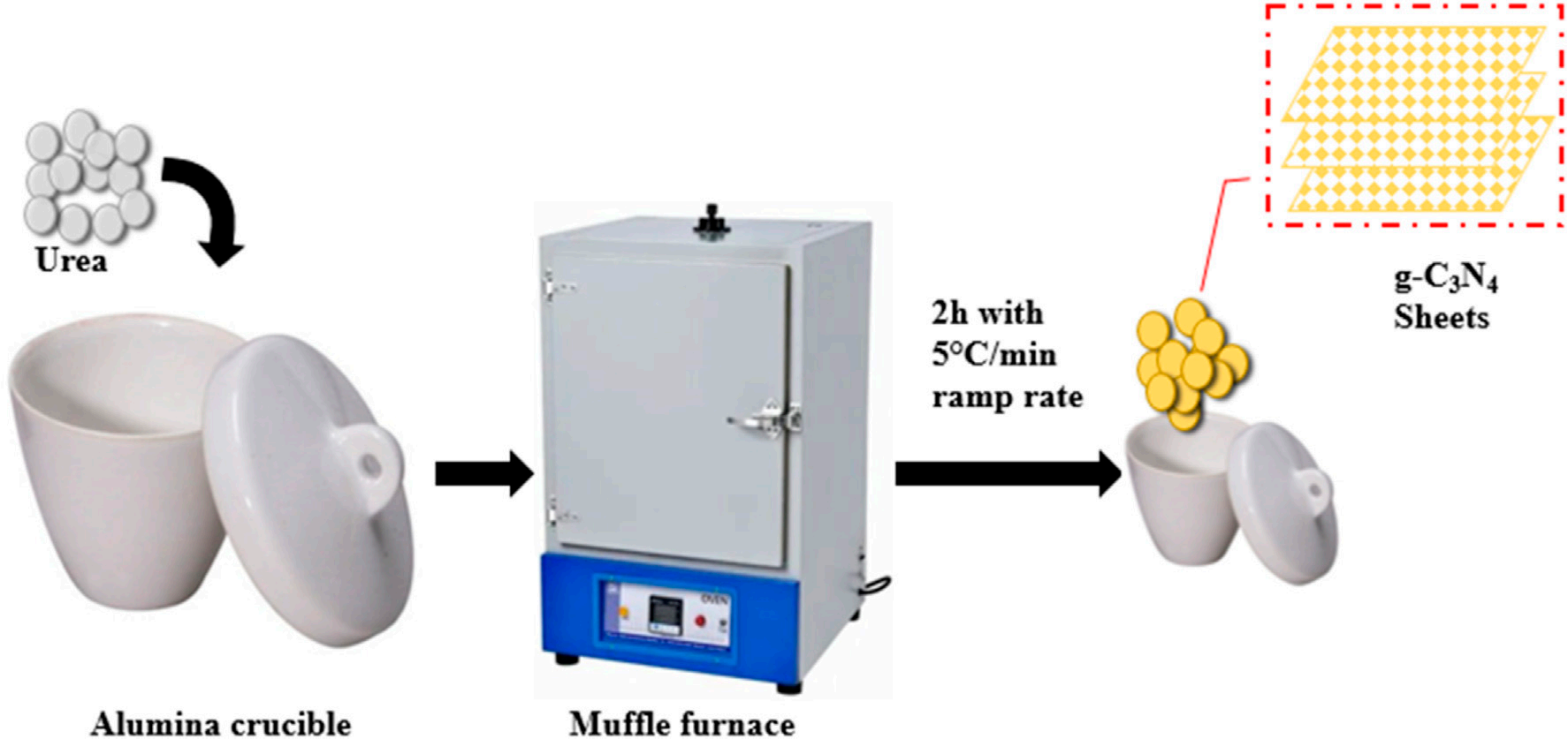
Synthesis of g-C_3_N_4_ by heating urea in a muffle furnace.

The direct decomposition method was used for the synthesis of g-C_3_N_4_. First, we added 100 g of analytical-grade CH_4_N_2_O and put a lid on the silica crucible. After that, we heated the crucible for 2 h in a muffle furnace to 550°C (Figure 1). The g-C_3_N_4_ was finally cleaned with C_2_H_6_O and DI H_2_O to eliminate impurities (Yao et al., 2018).

### 2.3 Synthesis of g-C_3_N_4_/TiO_2_/POA

**FIGURE 2:**
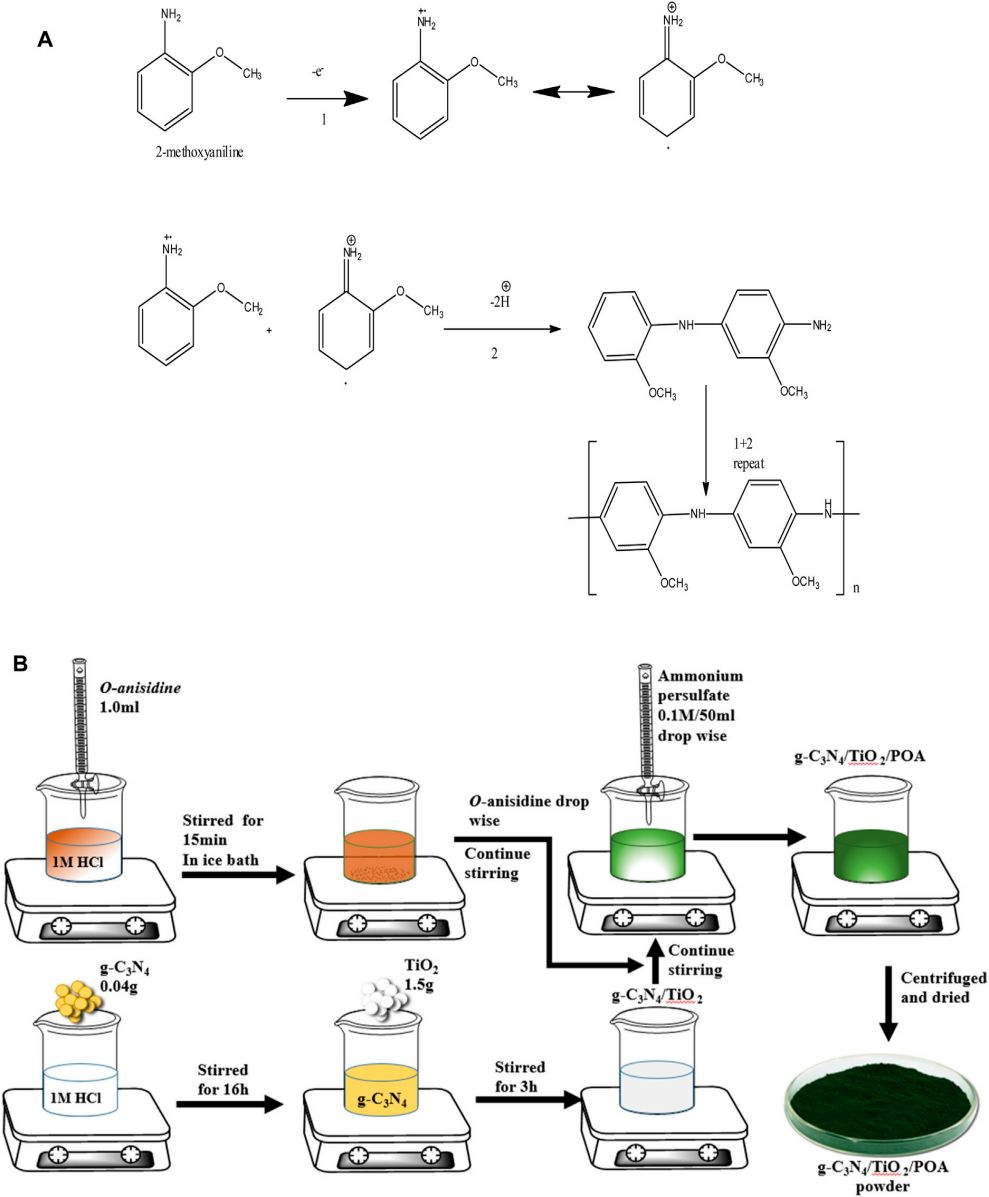
**(A)** Polymerization scheme of o-anisidine for the synthesis of polyorthoanisidine (POA). **(B)** Synthesis of the ternary composite g-C_3_N_4_/TiO_2_/POA.

The g-C_3_N_4_/TiO_2_/POA composite was synthesized using an *in-situ* chemical oxidative polymerization process. After adding 0.04 g of g-C_3_N_4_ to a 1.0M HCl solution, the mixture was agitated for 16 h. After that, 1.5 g of TiO_2_ was added, and stirring continued for 3 h. Then, in another beaker, 1 mL of o-anisidine was added dropwise to a 0.1M HCl solution and stirred at 200 rpm for 15 min in an ice bath. After adding the TiO_2_ solution, the aniline solution was agitated for 15 minutes. Oxidant ammonium persulfate (0.1M, 50 mL) was introduced dropwise and continuously stirred during polymerization. A bluish precipitate formed after 16 h of stirring at 200 rpm. The mixture was then filtered, thoroughly cleaned with acetone and water, and dried at 70°C (Figure 2B). The total yield obtained was 1.57 g (80.9%) composite. POA was synthesized using 1 mL of o-anisidine and a similar process without g-C_3_N_4_ or TiO_2_, yielding 0.4 g of POA (Figure 2A). It was found that the overall yield of the g-C_3_N_4_/TiO_2_/POA composite decreased by about 19.1%. The decrease in the yield percentage could result from some material being lost during the washing process.

### 2.4 Characterization

FTIR (Spectrum 2, Perkin Elmer, Waltham, MA, United States), XRD (D8 Advance Bruker, Vancouver, BC, Canada), and SEM (Cube II EmCrafts, Gyeonggi, Republic of Korea) were used to analyze the synthesized composite (g-C_3_N_4_/TiO_2_/POA). The functional groups of the composite (g-C_3_N_4_/TiO_2_/POA) were examined using FTIR. The crystallinity of the composite (g-C_3_N_4_/TiO_2_/POA) was investigated using XRD. Finally, SEM revealed the shape and homogenous dispersion of the composite.

### 2.5 Test of photocatalytic activity

**FIGURE 3:**
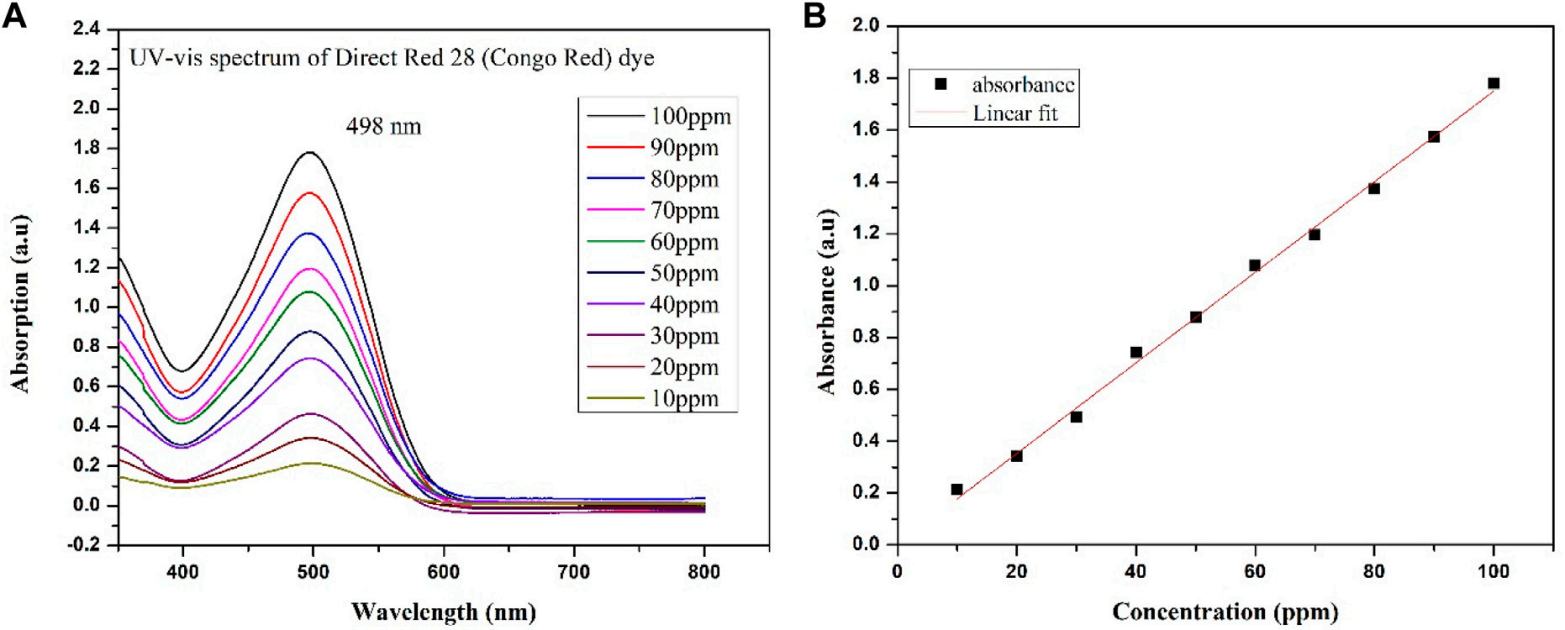
**(A)** UV-Vis spectra of different concentrations of Direct Red 28 dye. **(B)** Linear fit for the concentration of Direct Red 28 dye.

Anatase TiO_2_, g-C_3_N_4_, and g-C_3_N_4_/TiO_2_/POA were evaluated for their ability to degrade Direct Red 28 under ultraviolet light. First, a 150 mL dye solution with a defined pH and concentration was mixed with a predetermined quantity of catalyst. Before exposing the Direct Red 28 solution to UV light, the Direct Red 28 was adsorbed on the produced photocatalyst for 1 h in the dark to reach the adsorption-desorption equilibrium. Because the Direct Red 28 solution displayed a hue shift due to tautomerism below pH 4, the initial pH of the solution was maintained between pH 5 and 9. The pH of the Direct Red 28 solution was changed with NaOH and 0.1M HCl solutions. Furthermore, a range of 10–50 mg/L was used to test the impact of the starting Direct Red 28 dye concentrations on the photocatalytic process. The specimens were collected after constant intervals, cleared of the catalyst using a 0.22 μm syringe filter, and examined using the UV-visible spectrophotometer (UV 1800 Shimadzu) at 498 nm lambda max, as shown in (Figure 3). Eq. 1 illustrates the formula used to determine the photocatalytic degradation efficiency (E):

E=1−CCoor1−AAo (1)

#### 2.5.1 Kinetic analysis

The reaction pathway was identified using kinetic analysis. Literature has demonstrated that the expression of the Langmuir–Hinshelwood kinetic is compatible with a pseudo-first-order kinetic model for heterogeneous photocatalysis (Sun et al., 2009). Eq. 2 represents the Langmuir–Hinshelwood model in written form.

$$r={\text{kKC}}_{t}/1+{\text{KC}}_{t}\approx k_{\text{app}}C_{t}$$ (2)

K is the reactant adsorption constant, k_app_ is the reaction rate constant, and C_t_ is the dye concentration at a given time.

After integration, Eq. 2 can be written as Eq. 3:

$$\ln\backslash \left(C_{0}/C\right)=kt$$ (3)

The initial concentration of the dye solution is C_0_, and the rate constant is k.

## 3 Results and discussion

### 3.1 X-ray diffraction (XRD)

**FIGURE 4:**
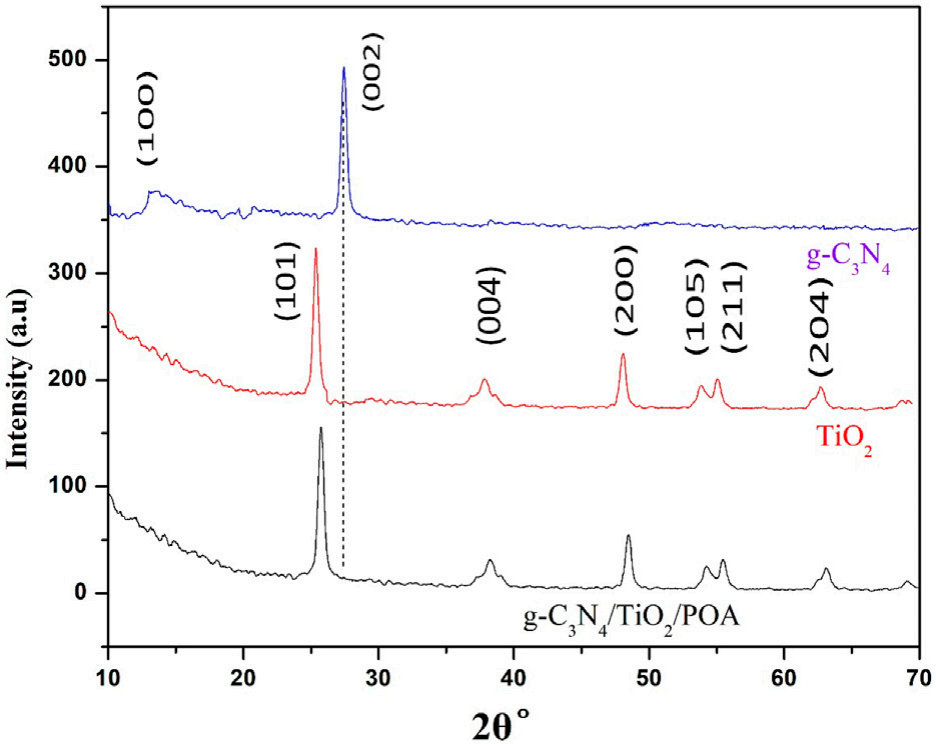
XRD of composite, TiO_2_, g-C_3_N_4_ and g-C_3_N_4_/TiO_2_/POA.

Figure 4 shows the XRD patterns of TiO_2_, GCN, and the composite to examine crystallinity, purity, and phase detection. The diffraction peaks matched standard diffraction data for TiO_2_ (Thamaphat et al., 2008). The XRD patterns of pure anatase TiO_2_ display sharp peaks of TiO_2_ at 2θ°≈25.3 (101), 38 (004), 48.05 (200), 53.8 (105), 55.09 (211), and 62.7 (204) (Kumar et al., 2016). Two prominent peaks can be seen in the XRD pattern for g-C_3_N_4_ at 2θ°≈ 12.9 (100) and 27.42 (002). All of the typical peaks of TiO_2_ 2θ°≈25.7 (101), 38.17 (004), 48.43 (200), 54.2 (105), 55.45 (211), and 63.06 (204) are visible in the XRD of the synthesized g-C_3_N_4_/TiO_2_/POA; however, the characteristic peak of GCN is not visible, which may be because there is inadequate GCN in the composite (Wang et al., 2017). There is no peak for POA due to its amorphous nature and small amount.

### 3.2 Fourier transform infrared spectroscopy (FTIR)

**FIGURE 5:**
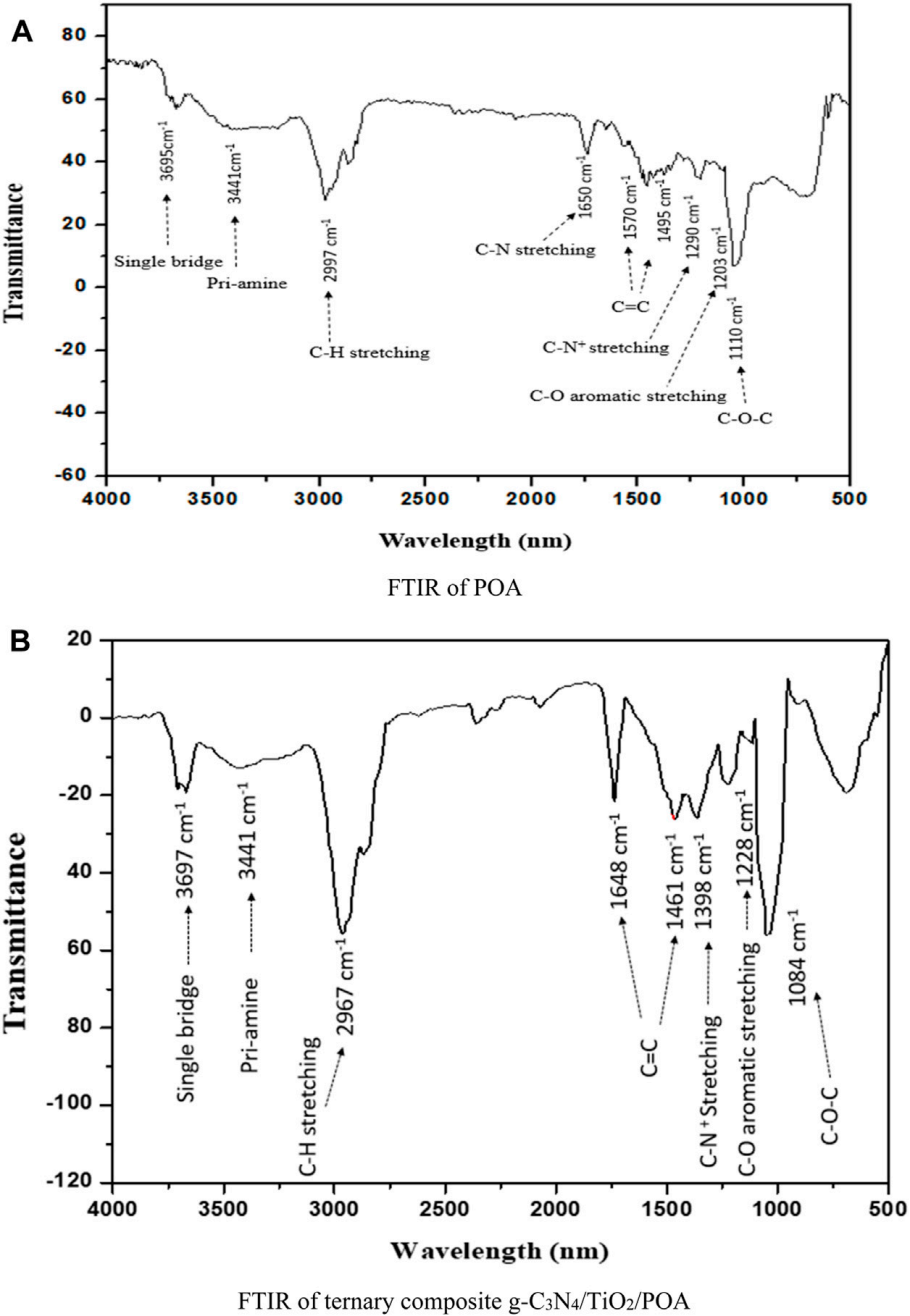
**(A)** FTIR of POA and **(B)** FTIR results of ternary composite g-C_3_N_4_/TiO_2_/POA.

Figure 5 shows the FTIR results of the POA and ternary composite g-C_3_N_4_/TiO_2_/POA. The chemical structure of pure POA is shown in Figure 5A, and the properties of the POA in the produced g-C_3_N_4_/TiO_2_/POA composite photocatalyst are shown in Figure 5B. Both the synthesized composite g-C_3_N_4_/TiO_2_/POA and POA exhibit peaks at 1,110 cm^−1^ and 1,084 cm^−1^ for C-O-C ether, 1,648 cm^−1^ and 1,650 cm^−1^ for the C=N stretching group, and 1,570 cm^−1^, 1,495 cm^−1^, 1,565 cm^−1^, and 1,461 cm^−1^ for the C=C group quinoid and rings. The infrared peaks at 2,967 cm^−1^ and 2,997 cm^−1^ show the C-H stretching, while the peaks at 1,203 cm^−1^ and 1,398 cm^−1^ show the C-O aromatic stretching. The primary amine exhibits peaks at 3,441 cm^−1^ in both images due to the N-H stretching vibration of the amine functional group (Manea et al., 2019). New bands at 3,646 cm^−1^ in the FTIR of POA and 3,697 cm^−1^ in the FTIR of the composite appear due to the polymeric association of single bridge compounds. The networks of both the o-anisidine and the g-C_3_N_4_ unique absorption band are present in the g-C_3_N_4_/TiO_2_/POA composite.

### 3.3 Scanning electron microscope (SEM)

**FIGURE 6:**
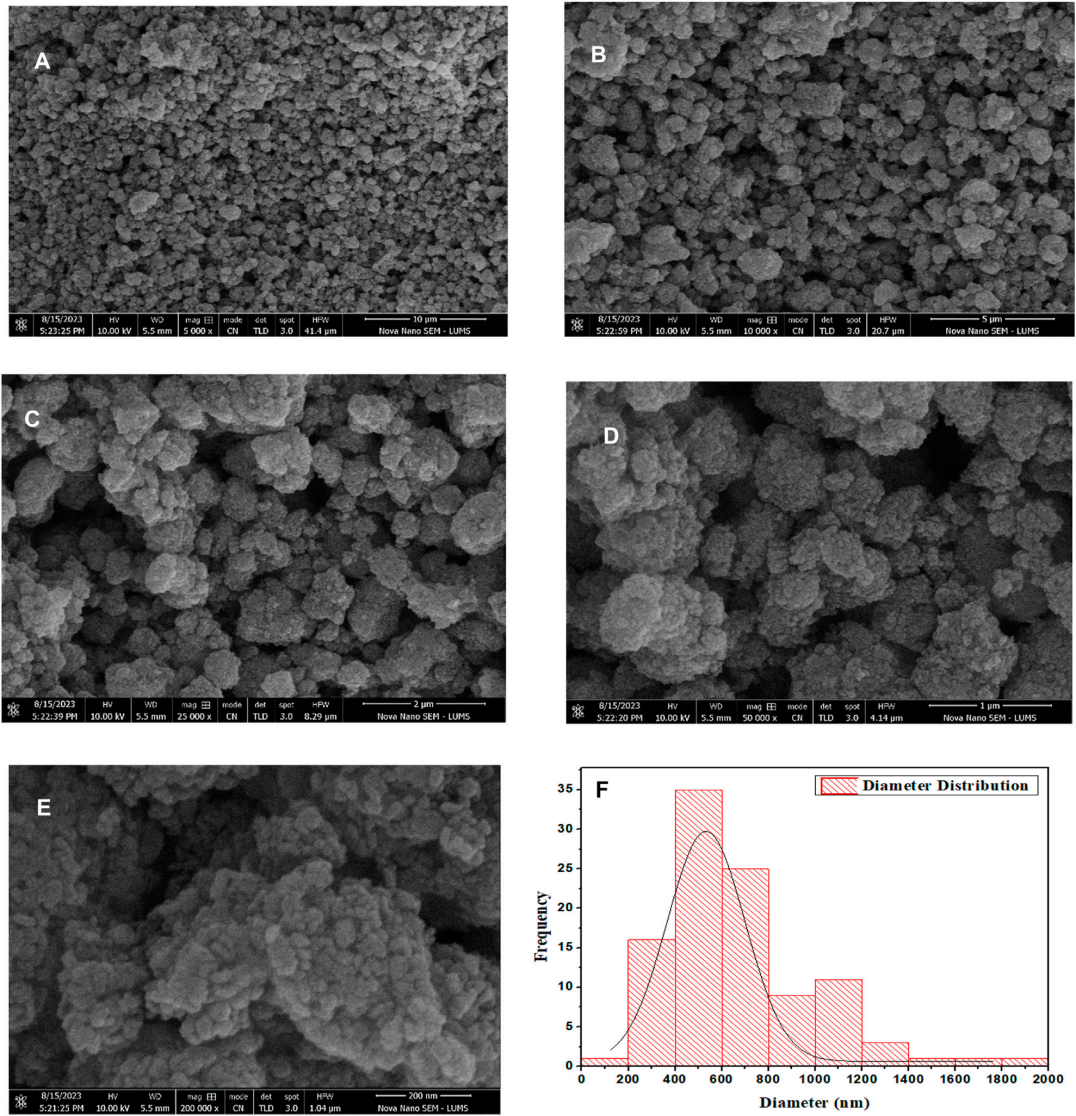
**(A–E)** SEM images of composite and **(F)** the histogram of composite particle size.

Figure 6 shows the SEM images of the produced g-C_3_N_4_/TiO_2_/POA ternary composite. The composite g-C_3_N_4_/TiO_2_/POA particles in the SEM picture are homogeneous (Figures 6A–E). TiO_2_ nanoparticles coated the surface of carbon nitride. The average particle size of the composite increased to 428 nm when TiO_2_ and POA were applied to the graphitic carbon nitride surface (Figure 6F). It was anticipated that TiO_2_ coatings would cause carbon nitride to take on a different shape, producing nearly spherical particles. The particle size of the composite was examined using the ImageJ digital processing program (Figure 6F).

### 3.4 Energy-dispersive X-Ray

**FIGURE 7:**
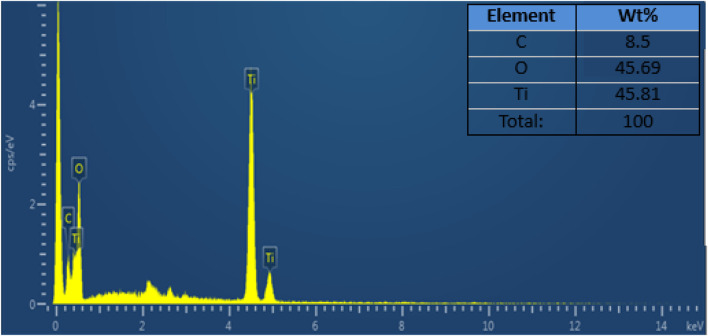
EDX spectra of g-C_3_N_4_/TiO_2_/POA.

Figure 7 shows the elemental composition of the composite that was investigated by the energy-dispersive X-ray (EDX) analytical technique. The EDX spectrum reveals that the weight percentages of the C, O, and Ti were 8.5%, 45.69%, and 45.81%, respectively. The spectrum supports the presence of C, O, and Ti particles in the composite (Talib et al., 2022).

### 3.5 Photocatalytic activity

**FIGURE 8:**
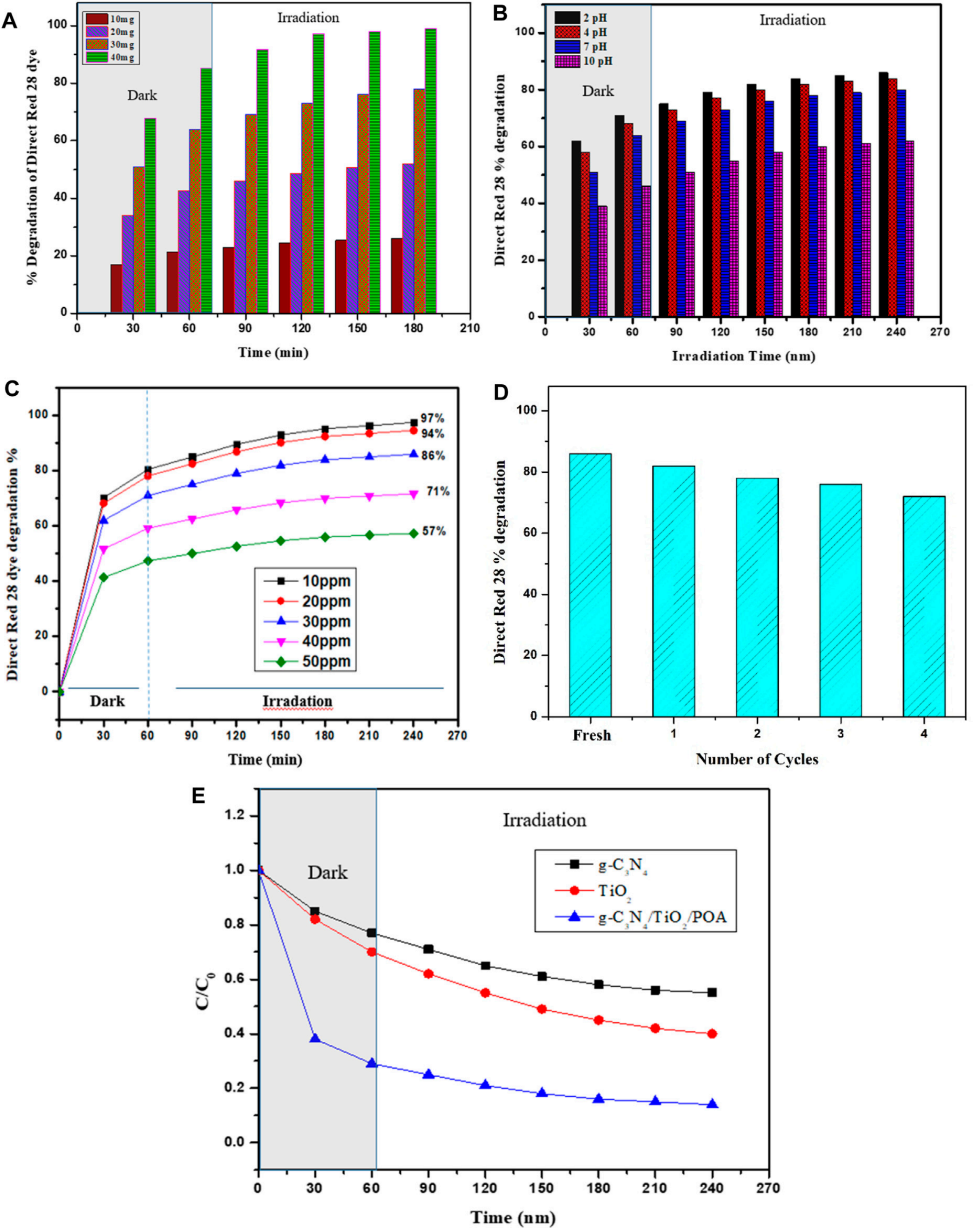
**(A)** The optimum catalyst dose was determined. **(B)** Effect of pH on the Direct Red 28 dye degradation by g-C_3_N_4_/TiO_2_/POA. **(C)** Effect of dye concentration on the Direct Red 28 dye degradation using g-C_3_N_4_/TiO_2_/POA. **(D)** Reusability study of g-C_3_N_4_/TiO_2_/POA composite. **(E)** Comparison of the degradation effects of TiO_2_, g-C_3_N_4_, and g-C_3_N_4_/TiO_2_/POA on Direct Red 28.

g-C_3_N_4_, TiO_2_, and the g-C_3_N_4_/TiO_2_/POA composite are shown in Figure 8E along with their photocatalytic characteristics. The findings demonstrate that, compared to g-C_3_N_4_ and TiO_2_, the g-C_3_N_4_/TiO_2_/POA composite exhibits better Direct Red 28 molecule degradation because of its reduced bandgap energy and improved separation of the e^−^/h^+^ pair. Furthermore, because the g-C_3_N_4_/TiO_2_/POA composite is hybrid and comprises a significant number of functional groups from g-C_3_N_4_, TiO_2_, and POA, Direct Red 28 molecules adsorb to it far more frequently than they do to g-C_3_N_4_ and TiO_2_. First, the active site on the g-C_3_N_4_/TiO_2_/POA composite rapidly interacts with the Direct Red 28 molecules in solution. Adsorbed Direct Red 28 molecules were degraded by photocatalysis in the following phase. When exposed to UV light, more dye concentration reduction occurs due to the absorption of UV light by TiO_2_. The degradation of the Direct Red 28 molecules onto the g-C_3_N_4_/TiO_2_/POA composite at various pH values is displayed in Figure 8B. The results indicate that the ideal conditions for Direct Red 28 molecule degradation are acidic (Miao et al., 2015). Based on the surface charge and contact between the g-C_3_N_4_/TiO_2_/POA composite and Direct Red 28 dye molecules, the degrading behavior of the g-C_3_N_4_/TiO_2_/POA composite can be understood. The g-C_3_N_4_/TiO_2_/POA composite was synthesized in an acidic environment. As a result, the g-C_3_N_4_/TiO_2_/POA composite contains protonated POA (emeraldine salt), which interacts electrostatically with the negatively charged Direct Red 28 molecules due to its net positive charge on the catalyst surface. As a result, a greater rate of photodegradation of Direct Red 28 dye molecules is seen at pH 2, 4, 6, and 7. Furthermore, in an essential medium, the g-C_3_N_4_/TiO_2_/POA surface has a surface charge that is neutralized by the hydroxyl ions of the basic medium. This results in a negatively charged g-C_3_N_4_/TiO_2_/POA surface that repels the anionic Direct Red 28 molecules. Furthermore, the fundamental requirement lowers the hydroxyl radicals’ potential for oxidation created during the photocatalysis reaction.

#### 3.5.1 Optimization of catalyst dose

Figure 8A shows the graph between time and % degradation for different catalyst dose concentrations. Photocatalytic degradation is enhanced by increasing the amount of catalyst; nevertheless, an ideal catalyst dose is required to optimize other parameters. Thus, to optimize catalyst dosage, 150 mL of a 30 ppm Direct Red 28 dye solution was degraded at pH 7 for 240 min using 10–40 mg of the synthesized composite g-C_3_N_4_/TiO_2_/POA. The % degradations by 10 mg, 20 mg, 30 mg, and 40 mg of the composite in 180 min shown in Figure 8A are 26%, 52%, 78%, and 95%, respectively. Hence, the 10 mg and 20 mg doses are too low to degrade dye quickly. The 40 mg dose shows a 95% reduction in dye concentration after only 60 min in darkness when adsorption equilibrium is achieved and is too large to examine the photocatalytic degradation mechanism of the g-C_3_N_4_/TiO_2_/POA catalyst. Therefore, 30 mg is a better dosage for analyzing other factors, including pH and dye concentration (Singh et al., 2013).

#### 3.5.2 pH effect on Direct Red 28 dye degradation

Figure 8B shows % degradation against time by adjusting the reaction mixture from pH 2.00 to 10 with a fixed Direct Red 28 dye concentration and optimized catalyst quantity. After 240 min, the study showed adjusting the pH of Direct Red 28 dye with NaOH and HCl at pH 10, 7, and 2 reduced the Direct Red 28 dye to 62%, 80%, and 86%, respectively. The catalyst works better at low pH for Direct Red 28 dye degradation because of the anionic character of the dye and the presence of two sulphonic groups. These groups quickly ionize in an acidic solution to produce soluble Direct Red 28 anions. Furthermore, g-C_3_N_4_/TiO_2_/POA composites have more protons to protonate the hydroxyl groups at lower pH levels. This creates an electrostatic attraction that pulls a small quantity of dye anion into the material. Because of their positive surface charges, Direct Red 28 anions quickly adsorbed on g-C_3_N_4_/TiO_2_/POA composites at low pH levels. However, additional negatively charged adsorbent sites resist the absorption of Direct Red 28 anions to the catalyst’s surface at higher pH values. Electrostatic repulsion prevents an anionic dye from being adsorbed to a negatively charged area of the adsorbent. As can be seen in Figure 8B, the results indicate that an acidic medium facilitates the Direct Red 28 dye degradation more than a basic medium.

#### 3.5.3 Effect of dye concentration

Figure 8C illustrates the % degradation of dye against time by varying dye concentration from 10 ppm to 50 ppm using a constant catalyst concentration of 30 mg of g-C_3_N_4_/TiO_2_/POA. Results show that at 30 ppm initial dye concentrations, 86% degradation was achieved at 240 min. As the initial dye concentration increased from 40 ppm to 50 ppm, the dye degradation decreased to 71% and 57%, respectively. In 240 min, the dye degradation increased from 86% to 97.5% due to the drop in initial dye concentration from 30 ppm to 10 ppm. This might be because when the concentration of the dye increases, less light reaches the surface of the catalyst, which lowers photocatalytic activity because fewer photocatalytic radicals with high initial dye concentration are produced (Tayeb and Hussein, 2015).

#### 3.5.4 Reusability of g-C_3_N_4_/TiO_2_/POA

Figure 8D illustrates the % dye degradation of 300 mL dye solution containing 30 ppm of Direct Red 28 dye at pH 2 against the four cycles of 60 mg g-C_3_N_4_/TiO_2_/POA. According to findings, 86% of the Direct Red 28 dye molecules were degraded in the fresh g-C_3_N_4_/TiO_2_/POA composite. However, after the fourth cycle, the percentage of degradation of Direct Red 28 dye decreased to 72%. The decrease in the photocatalytic performance of the g-C_3_N_4_/TiO_2_/POA composite can be attributed to either the internal structure of the catalyst or the adsorbed molecules of Direct Red 28 dye on active sites of the composite (Erdemoğlu et al., 2008). These findings showed that, in the given experimental circumstances, the g-C_3_N_4_/TiO_2_/POA composite is a stable catalyst. To ensure its recyclability, the g-C_3_N_4_/TiO_2_/POA composite was washed and dried at 60°C for 12 h after each cycle using deionized water.

#### 3.5.5 Kinetic analysis

**FIGURE 9:**
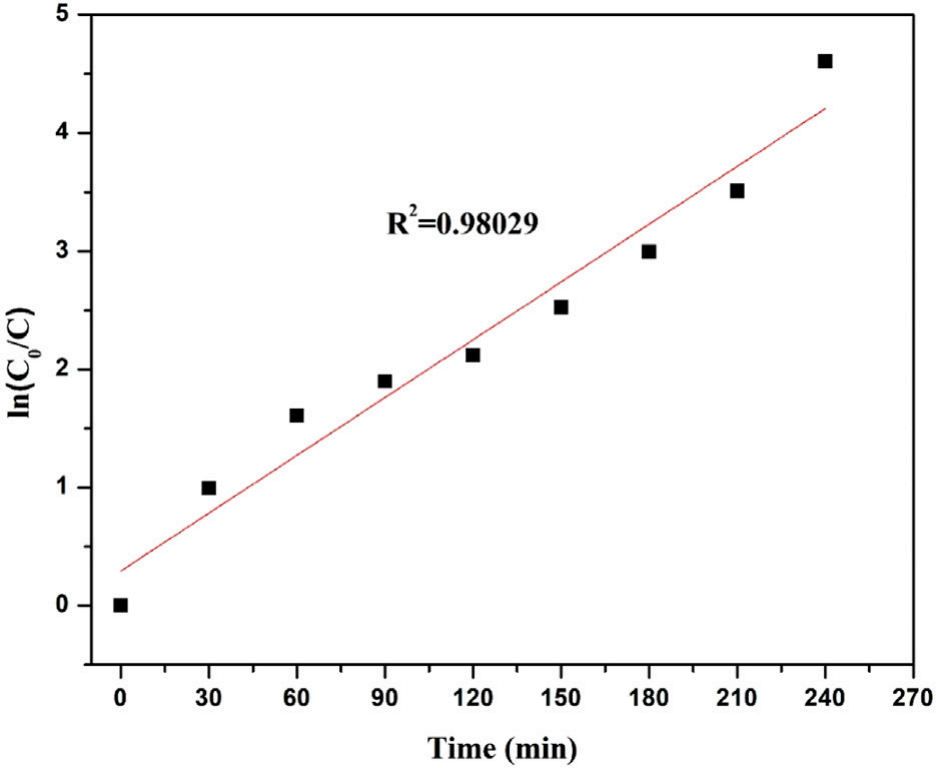
First-order kinetic model for the % degradation of Direct Red 28 by g-C_3_N_4_/TiO_2_/POA.

The straight line on the plot of ln C_0_/C vs. irradiation time t in Figure 9 shows that Direct Red 28 dye degradation follows pseudo-first-order kinetics. Because the regression coefficient (*R* ^2^) is 0.98029, which is near 1, it supports the L-H kinetic model and shows that the photocatalyst adheres to the pseudo-first-order kinetic model. Additionally, the regression coefficients for the zero- and second-order kinetics were found to be 0.79919 and 0.59317, respectively (see in Table 1), indicating that the *R* ^2^ for the first-order kinetics was closer to 1 than it was for the zero- and second-order kinetics, confirming that the response adheres to first-order kinetics.

**TABLE 1:** Correlation coefficients (*R* ^2^) for zeroth, first, and second-order kinetic models of photocatalytic reaction.

Kinetics	Correlation coefficient *R* ^2^
Zero-order kinetics: C_0_X = kt	0.79919
First-order kinetics: ln1/(1−X) = kt	0.98029
Second-order kinetics: X/C_0_(1−X) = kt	0.59317

## 4 Photocatalytic mechanism

**FIGURE 10:**
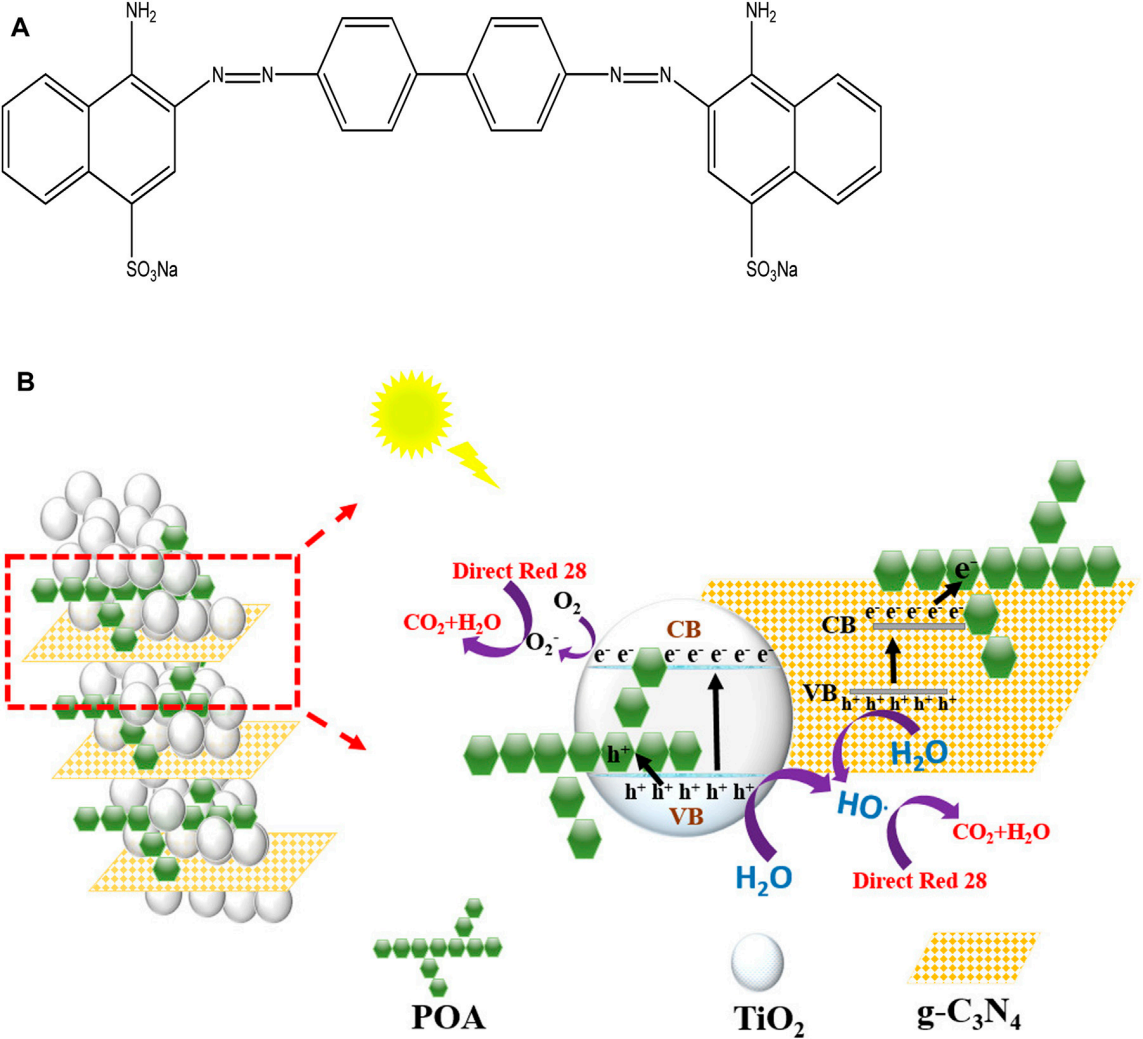
**(A)** Structure of Direct Red 28 dye. **(B)** Proposed photocatalytic mechanism of g-C_3_N_4_/TiO_2_/POA degradation of Direct Red 28.

Figure 10 explains the enhanced photocatalytic activity of the g-C_3_N_4_/TiO_2_/POA composite, including effective UV light absorption and electron-hole pair separation. Upon UV light irradiation, the electron/hole pairs get separated in both TiO_2_ and g-C_3_N_4_. The holes in the g-C_3_N_4_ valence band recombine with the electrons in the TiO_2_ conduction band. According to Li et al. (2015), this phenomenon stops electrons and holes from recombining. Furthermore, the POA surface also accepts the holes that are produced. Thus, the electrons stayed in the g-C_3_N_4_ conduction band, combining with oxygen to generate reactive superoxide radicals (O^2−^), which can damage the dye molecule directly (Figure 10). Additionally, some of the O_2_ and H_2_O react and produce OH^•^. According to Yu et al. (2000), the holes in the POA surface and the valence band of TiO_2_ react with H_2_O to produce additional OH• radicals. Overall, this composite reduces the recombination rate of e−/h+ pairs and degrades more efficiently than single materials like TiO_2_ and g-C_3_N_4_.

## 5 Conclusion

The ternary composite of g-C_3_N_4_/TiO_2_/POA was synthesized to reduce the e^−^/h^+^ recombination rate in the photocatalytic degradation of Direct Red 28. XRD, FTIR, SEM, and EDX were used to evaluate the characteristics of the produced g-C_3_N_4_/TiO_2_/POA composite. The composite (g-C_3_N_4_/TiO_2_/POA) was assessed as an active photocatalyst for the degradation of Direct Red 28 dye under UV light. The result showed that a 30 mg/L dose of the synthesized ternary g-C_3_N_4_/TiO_2_/POA composite in 240 min at pH 2 showed 86% degradation of Direct Red 28 dye, compared to g-C_3_N_4_ and TiO_2_, which showed 45% and 60% degradation, respectively. The synthesized ternary g-C_3_N_4_/TiO_2_/POA composite had greater photocatalytic activity than g-C_3_N_4_ and TiO_2_. After four consecutive cycles, the utilized composite showed 79% degradation of Direct Red 28, demonstrating the stability and effectiveness of the g-C_3_N_4_/TiO_2_/POA photocatalyst. First-order kinetic model analysis was used to examine the experimental data, which showed that Direct Red 28 dye degradation follows pseudo-first-order kinetics. Our finding shows that the ternary composite of g-C_3_N_4_/TiO_2_/POA is appropriate for changing the band gap, reducing the rate of e^−^/h^+^ pair recombination, and enhancing photocatalytic degradation of Direct Red 28 dye molecules. All reactions were run using an optimized catalyst dose. Overall, the Direct Red 28 dye degradation process using the g-C_3_N_4_/TiO_2_/POA composite was non-toxic, cost-effective, and environment-friendly in terms of both synthesis and photocatalytic activity.

## Data Availability

The raw data supporting the conclusion of this article will be made available by the authors, without undue reservation.
